# Effect of a Financial Education and Coaching Program for Low-Income, Single Mother Households on Child Health Outcomes

**DOI:** 10.3390/healthcare12020127

**Published:** 2024-01-05

**Authors:** Kevin T. Fuji, Nicole D. White, Kathleen A. Packard, Julie C. Kalkowski, Ryan W. Walters

**Affiliations:** 1 Department of Pharmacy Sciences, School of Pharmacy and Health Professions, Creighton University, Omaha, NE 68178, USA; 2 Department of Pharmacy Practice, School of Pharmacy and Health Professions, Creighton University, Omaha, NE 68178, USA; 3 Financial Hope Collaborative, Heider College of Business, Creighton University, Omaha, NE 68178, USA; 4 Department of Clinical Research and Public Health, School of Medicine, Creighton University, Omaha, NE 68178, USA

**Keywords:** child and adolescent health, social determinants of health, financial education, single mothers, low income

## Abstract

The financial difficulties of parents have a negative impact on the health of their children. This problem is more pronounced in single mother families. There is limited research on low-income, single mothers and how interventions to help them address financial difficulties may also benefit their children. The purpose of this study was to evaluate the effect of a year-long financial education and coaching program on school absenteeism and health care utilization of children in employed, low-income, single mother households. This was a post hoc analysis of the Finances First study, a randomized controlled trial conducted in 2017–2020 examining the impact of a financial coaching and education program on economic stability and health outcomes in 345 low-income, single mothers. Either generalized estimating equations (GEEs) or generalized linear mixed models (GLMMs) were used to account for relationships between participants. For the continuous outcomes of child absenteeism, physician visits, emergency room visits, and hospitalization days, a linear mixed-effects model was used. The Finances First study demonstrated improvements in various financial strain measures. Compared to the control group, children of intervention group participants experienced 1 fewer day of school absence (*p* = 0.049) and 1 fewer physician visit (*p* = 0.032) per year, but no impact was seen on emergency room visits (*p* = 0.55) or hospitalizations (*p* = 0.92). Addressing social determinants of health in parents is necessary for improving child health outcomes.

## 1. Introduction

A focus on child health is critical to both the short- and long-term success of communities [1]. However, many factors can lead to sub-optimal child health outcomes [2]. Some of the more prominent factors include parental difficulties related to social determinants of health (SDOHs). SDOHs are defined by the Centers for Disease Control and Prevention as, “the nonmedical factors that influence health outcomes. They are the conditions in which people are born, grow, work, live, and age, and the wider set of forces and systems shaping the conditions of daily life” [3]. A recent study examining children receiving primary care at a large hospital-based pediatric institution found that 12% of their families reported at least one social need. Children whose families had social needs were more likely to have either a hospital or emergency room visit compared to children whose families reported no social needs [4].

A common SDOH experienced by parents is financial difficulties, with an estimated 38% of children in the United States (U.S.) under the age of 18 residing in a low-income family [5]. Parental financial stress negatively impacts financial behaviors, personal relationships, and health behaviors [6]. These negative parental outcomes are passed on to children in multiple ways, including increased problematic behaviors in school, absence from school, poor health and well-being, and overall stunted development, which can have lifetime impacts [7,8] School attendance and education is one way in which economic hardships as a child can be mitigated as an adult. However, 13% of all children in the U.S. are chronically absent from school, defined as missing more than 10% or approximately 18 days of school per year [9]. Chronic absenteeism is linked to not only poor academic performance, but also health outcomes [9].

All of these SDOH-related problems are exacerbated in single mother households due to their dual roles as a primary caregiver to their children while simultaneously serving as the lone (or major) source of income. However, there is limited research examining how interventions designed to support single mothers address financial difficulties result in changes in child absenteeism and health. Early work examining the impact of a standardized financial coaching and education intervention that was utilized in the present study revealed that children of low-income, single mothers achieved a decrease in body mass index (BMI), suggesting the potential for health benefits to accrue in children despite an intervention targeted towards their mothers [10].

### 1.1. Financial Success Program (FSP)

The FSP is housed in the Financial Hope Collaborative within the Creighton University Heider College of Business. The FSP was established in 2009 and has two sequential components, a nine-week set of group classes followed by one year of one-on-one financial coaching. There are three primary focus areas for the intervention: addressing the immediate financial issues of participants, learning and practicing financial skills and behavior change, and supporting the development of decision-making strategies to foster long-term financial confidence. The core topics for the nine-week group classes are the psychology of money, money management, predatory lending, credit reports, understanding utilities, understanding insurance and retirement, ways to save, goal setting, and financial products [11]. Participants are then paired with a coach who provides tailored support that builds off the core topics in the group classes but is individualized to the needs of the participant. Single mothers have been the target population of the FSP, and the curriculum was designed by single mothers specifically for financial issues they face in daily life. More in-depth information about the intervention content has been previously published [12].

### 1.2. Conceptual Frameworks

This study was built on two conceptual frameworks, the Limited Resource Model of Self-Control and the Family Stress Model. The Limited Resource Model of Self-Control posits that each individual has a finite amount of self-control resources and that once those resources are depleted, decision making becomes more difficult. Limited financial resources force individuals into more frequent difficult decisions, depleting self-control resources more rapidly and ultimately not leaving enough for individuals to utilize for health care decision making, supporting the uptake of unhealthy behaviors [13]. This has sometimes been referred to as a “bandwidth tax” and has been provided as a reason why interventions that are designed to promote healthy behaviors are often unsuccessful [14]. Addressing financial difficulties will ease financial strain, leaving more self-control resources available to focus on the health of both the individual and their children.

The Family Stress Model posits that financial strain has a negative parental impact, which ultimately results in poorer outcomes for children [15]. Combined with the Limited Resource Model of Self-Control, addressing financial difficulties will ease financial strain, leaving more self-control resources available to focus on both the health of the individual and their children.

The purpose of this study was to evaluate the effect of the FSP on school absenteeism and health care utilization of children in employed, low-income, single mother households.

## 2. Materials and Methods

This study utilized a post hoc analysis of the Finances First study, which was conducted from April 2017 to August 2020. Finances First was a randomized controlled trial examining the impact of the FSP intervention on economic stability and health outcomes in low-income, single mothers. The primary endpoint of the Finances First study was the proportion of overweight and obese participants who had a ≥5% reduction in weight. Secondary outcomes included financial stress, health care utilization, and smoking status. The outcomes of interest for this post hoc analysis were school absenteeism, physician visits, emergency room visits, and hospital days for children during the one-year study period. The study was approved by the Creighton University Institutional Review Board (IRB #1011656), and informed consent was obtained from all participants.

### 2.1. Study Population and Recruitment

Participants were recruited from the Omaha metropolitan area using a convenience sampling approach through word of mouth, flyers, and personal and community contacts. Inclusion criteria were self-identified single mothers, aged 19–55 years old, English- or Spanish-speaking, and with current employment but an annual income <200% of the federal poverty level. The U.S. Department of Health and Human Services establishes the poverty guidelines each year, which takes into account family size and household income. In 2017, when the Finances First study began enrollment, a family of two with an annual income less than USD 32,480 or a family of four with an annual income less than USD 49,200 would be categorized as having an income <200% of the federal poverty level [16]. The poverty guidelines are used as an eligibility criterion by numerous federal programs, such as Medicaid and the Supplemental Nutrition Assistance Program. Most federal programs use incomes <200% of the federal poverty level as thresholds for eligibility, and therefore, this level was selected as an inclusion criterion for the study to estimate financial instability or financial stress. Exclusion criteria were individuals who were pregnant or had active plans to become pregnant, actively engaged in substance abuse, or living in a domestic violence situation. Pregnant or active plans for pregnancy was a criterion as the intent of the study was to evaluate the impact of the FSP intervention on health outcomes of women. This would minimize pregnancy-related effects on health (e.g., changes in body weight, blood pressure, blood glucose, and cholesterol levels).

To account for Spanish-speaking women, the FSP intervention was adapted by recruiting single mothers from similar cultural backgrounds to serve as educators and financial coaches. Additionally, all FSP educational materials were converted from English to Spanish. Information about remittance was also included for Spanish speakers.

Individuals were randomized to either the intervention group, who engaged in the FSP, or the control group, who received no financial education or training. All individuals in the control group were given the opportunity to receive the FSP intervention upon study conclusion. A 1:1 randomization process was generally used. However, given the convenience sampling approach and to minimize cross-group contamination, women who were referred to the study by an existing participant were assigned to the same group as the referrer. A total of 32 participants were enrolled through referral of an existing participant, and 27 of these individuals were assigned to the intervention group.

A total sample size of 246 participants (123 in each group) was needed to achieve 80% power using a two-sided alpha of 0.05 to detect a 30% difference in the number of participants achieving weight loss of ≥5%. Based on a pilot study in which 70% of women were overweight or obese at baseline, and accounting for a 20% dropout rate, a total of 440 participants (220 in each group) were needed to maintain statistical power. At the conclusion of the Finances First study, there were a total of 345 participants (161 in the control group and 184 in the intervention group).

### 2.2. Data Collection

Data from the Finances First study were collected at baseline and the end of study visit at 12 months. In addition to demographic data (age, race/ethnicity, education level, insurance status) and common biometric data (body mass index, systolic and diastolic blood pressure, a complete lipid panel, and hemoglobin A1c), the validated Family Economic Strain Scale (FESS) was used to measure perceived financial stress, and a researcher-developed questionnaire was used to measure the impact of financial stress on sleep, health, personal relationships, and ability to work [17]. Data for this post hoc analysis were collected through monthly phone calls to participants during the 12-month Finances First study period. At the end of each month, participants were called and asked how many times in the previous month their child missed school, had a physician visit, went to the emergency room, or spent time in the hospital.

### 2.3. Data Collection

Data for the Finances First study were analyzed in three ways: intention to treat (ITT), a modified ITT, and per protocol [18]. This post hoc analysis utilized the ITT sample. This was done to maximize sample size as it included all participants who enrolled in the study and were randomized to the intervention group (including those who did not complete any FSP group classes). Categorical variables were analyzed descriptively and presented as frequency counts and percent. Continuous variables were analyzed descriptively and presented as means along with associated minimum and maximum values. The ITT analysis used either generalized estimating equations (GEEs) or generalized linear mixed models (GLMMs) to account for the correlation inherent to the referrer–referee relationship. The choice of analytic approach was based on interpretation of the data-scale values. For the GEE model, effects represented the average across all study participants compared to the GLMM, which was woman-specific. Both models used either a two- or three-level sampling structure depending on whether repeated observations from the same woman were included in the analysis [19]. For the continuous outcomes in this post hoc analysis, a linear mixed-effects model was used, which accounted for the number of children per participant. Analyses were conducted using SAS v9.4 (SAS Institute, Inc., Cary, NC, USA) with a significance level of *p* ≤ 0.05.

## 3. Results

A summary of overarching demographics from the Finances First study is provided in Table 1. Briefly, both the intervention and control groups were statistically similar, with a mean age of 35 years, over 50% being Black or African American, most having at least some college education (with over a third in each group being a college graduate), and the majority having commercial health insurance coverage at baseline. Additionally, participants in both groups had a mean of 2 children (min: 1, max: 3 in the control group and min: 1, max: 2 in the intervention group) with an average age of 10 years in the control group (min: 5, max: 14) and 9 years (min: 6, max: 13) in the intervention group. A more detailed breakdown of participant demographics and primary study outcomes has been previously published [18].

### 3.1. Relevant Primary Study Outcomes

At study conclusion, financial strain as measured by the FESS decreased to a greater degree in the intervention group compared to the control group (difference of 3.67, 95% CI: 1.60 to 5.75, *p* < 0.001). The odds of reporting negative effects of financial strain on relationships was 32% lower in the intervention group compared to the control group (*p* = 0.010), the odds of reporting negative effects of financial strain on health was 28% lower in the intervention group compared to the control group (*p* = 0.021), and the rate of participants failing to seek health care due to cost was 13% lower in the intervention group compared to the control group (*p* = 0.030).

### 3.2. Child Health Outcomes

Children whose mothers were in the intervention group had on average one fewer missed day of school (*p* = 0.049) and one fewer physician visit (*p* = 0.032) during the study period compared to children whose mothers were in the control group. There was no difference between the intervention and control group for the average number of emergency room visits (*p* = 0.55) or hospitalization days (*p* = 0.92). Table 2 displays a more detailed breakdown of the child health outcomes across the two groups.

## 4. Discussion

Findings from this post hoc analysis of the Finances First study demonstrated a positive impact for children on school absenteeism and physician visits over a year-long study period. School absenteeism has been associated with numerous negative outcomes not just related to academic performance and education but also long-term impacts on income and health [20]. Thus, decreasing levels of absenteeism, even by a day per academic year, can potentially yield significant short- and long-term benefits for children. While the reason for having one less physician visit per year was not captured, given the positive impacts of the FSP intervention for mothers, it is highly likely that their children also experienced better health, resulting in less of a need for physician visits [21]. A more longitudinal look at these impacts is needed to better understand if positive child outcomes are sustained and potentially increase as mothers’ financial behaviors become concretized. Regardless, these results suggest that addressing financial stress at the parental level can indirectly improve child health and makes a case for multi-faceted interventions that target both mother and child. Although no change was detected in emergency room visits or hospitalization days, the analysis was likely underpowered to detect differences in these less frequently experienced events.

Study findings suggest that focusing on only traditional health needs is insufficient for impacting the health of children in a low-income, single mother household. There are ongoing efforts to solicit information about SDOHs, document this information in the patient’s electronic health record, and utilize this information in the treatment of patients [22]. This is occurring across multiple health care settings, including during both primary care family appointments and pediatric visits [23]. Standardizing SDOH screening at every health care visit, including well-child visits, is an important first step to addressing these issues.

The Finances First study was one of the first to examine the impact of a financial education and coaching intervention on health outcomes. There is a need to develop, implement, and test SDOH interventions (both practice- and policy-based) in more holistic ways that connect to health outcomes. For example, one study utilized patient navigators to address family social needs and found that this strategy resulted in a decreased risk of hospitalization for children [24]. From a policy perspective, the expansion of the 2021 Child Tax Credit decreased child poverty, reduced food insecurity, and improved parents’ health, although the impact on child health has yet to be fully examined [25].

While the FSP intervention integrated some adaptations to account for Spanish-speaking individuals, more work is needed to understand how cultural backgrounds and traditions are optimally addressed within similar types of interventions addressing not only finances but other SDOH [26].

Finally, all of this work should be considered within the context of single mothers. There is a general lack of understanding of the differential impact of SDOH on this sub-population, which is key to guiding future efforts to address these ongoing problems [27].

### Limitations

There were several limitations to this study. A selection bias may have been present as women who enrolled in the Finances First study may have been more inherently motivated and prepared to make positive changes in both their own and their children’s lives.

The sample was also highly educated, with over a third of participants in each group being a college graduate. While this is a limitation, given the positive study findings, it challenges the traditional biases/stereotypes that highly educated individuals do not struggle with financial literacy and would not benefit from this type of intervention. Future research with a more diverse sample of educational backgrounds is needed to expand on these initial findings.

The intervention targeted single mothers, meaning that findings may not be generalizable to a wider population, including more traditional two-parent households or those outside of the operational definition of low-income used in this study (>200% of the federal poverty level).

Finally, given that the child health outcome data were self-reported and not objectively validated by the researchers, there was the potential for recall bias and inaccurate reporting. While monthly phone calls likely reduced this bias, more frequent contact (e.g., weekly phone calls) may have facilitated the most accurate level of reporting. Also, there were no pre-intervention data collected for the child outcomes, which limits understanding of the intervention’s impact.

## 5. Conclusions

A financial education and coaching intervention designed for low-income, single mothers not only decreased financial strain for mothers but led to fewer days of missed school and fewer physician visits for their children. Addressing SDOH such as economic stability supports a more holistic approach to care that recognizes the impact these factors have not only on the health of the individual but also their dependents, such as children. Future research should continue looking broadly at the sphere of impact resulting from these types of SDOH interventions.

## Figures and Tables

**Table 1:** Key baseline demographic variables for the study sample.

	Control (*n* = 161)	Intervention (*n* = 184)
Age, mean (SD)	35.1 (7.8)	34.6 (7.6)
Baseline BMI, score (SD)	33.8 (9.3)	33.3 (8.6)
Race and ethnicity, *n* (%)		
Non-Hispanic White	48 (29.8)	39 (21.2)
Black/African American	87 (54.0)	101 (54.9)
Latina/Hispanic	21 (13.0)	30 (16.3)
Other	5 (3.1)	14 (7.6)
Education, *n* (%)		
Some high school	10 (6.3)	14 (7.7)
High school graduate	14 (8.8)	26 (14.2)
Some college	77 (48.1)	81 (44.3)
College graduate	59 (36.9)	62 (33.9)
Baseline health insurance, *n* (%)		
None	44 (27.3)	53 (28.8)
Medicaid	48 (29.8)	50 (27.2)
Medicare	3 (1.9)	2 (1.1)
Commercial	68 (42.2)	82 (44.6)
Government	0 (0)	2 (1.1)

**Table 2:** Comparison of child absenteeism and health utilization outcomes.

	Control (*n* = 161)	Intervention (*n* = 184)
Missed school days, mean (minimum, maximum)	4 (2, 9)	3 (2, 6)
Physician visits, mean (minimum, maximum)	3 (2, 5)	2 (1, 4)
Emergency room visits, mean (minimum, maximum)	1 (0.5, 1)	1 (0.5, 2)
Hospital days, mean (minimum, maximum)	1 (0.5, 1)	1 (0.3, 1)

## Data Availability

The data presented in this study are currently being used in a longitudinal follow-up dataset and are not publicly available due to restrictions.
